# A Nutrigenomic View on the Premature-Aging Disease Fanconi Anemia

**DOI:** 10.3390/nu16142271

**Published:** 2024-07-15

**Authors:** Eunike Velleuer, Carsten Carlberg

**Affiliations:** 1 Department for Cytopathology, Heinrich-Heine-University Düsseldorf, D-40225 Düsseldorf, Germany; velleuer@uni-duesseldorf.de; 2 Department for Pediatric Hemato-Oncology, Helios Children’s Hospital, D-47805 Krefeld, Germany; 3 Institute of Animal Reproduction and Food Research, Polish Academy of Sciences, PL-10-748 Olsztyn, Poland; 4 School of Medicine, Institute of Biomedicine, University of Eastern Finland, FI-70211 Kuopio, Finland

**Keywords:** Fanconi anemia, premature aging, lifestyle choices, squamous cell carcinoma, immunocompetence, nutrigenomics

## Abstract

Fanconi anemia, a rare disorder with an incidence of 1 in 300,000, is caused by mutations in *FANC* genes, which affect the repair of DNA interstrand crosslinks. The disease is characterized by congenital malformations, bone marrow failure within the first decade of life, and recurrent squamous cell carcinomas of the oral cavity, esophagus, and anogenital regions starting around age 20. In this review, we propose that Fanconi anemia should be considered a premature-aging syndrome. Interestingly, the onset and severity of the life-limiting clinical features of Fanconi anemia can be influenced by lifestyle choices, such as a healthy diet and physical activity. These factors shape the epigenetic status of at-risk cell types and enhance the competence of the immune system through nutritional signaling. Fanconi anemia may serve as a model for understanding the aging process in the general population, addressing research gaps in its clinical presentation and suggesting prevention strategies. Additionally, we will discuss how the balance of genetic and environmental risk factors—affecting both cancer onset and the speed of aging—is interlinked with signal transduction by dietary molecules. The underlying nutrigenomic principles will offer guidance for healthy aging in individuals with Fanconi anemia as well as for the general population.

## 1. Introduction

Aging is a natural process that occurs at different rates for each individual [1]. It is characterized by the accumulation of molecular and cellular damage, which leads to dysfunction in cells, tissues and organs, ultimately weakening the entire body. The primary hallmark of aging is genome instability, evidenced by the accumulation of point mutations, insertions and deletions in the genomes of all body cells [2]. The overlap between the hallmarks of aging and those of major non-communicable diseases, such as cancer, diabetes, cardiovascular diseases (CVD) and neurodegenerative disorders, provides the mechanistic basis for understanding why these diseases are age-related [3].

The maximum human lifespan is around 110–120 years, but only a few individuals achieve this age [4]. Studies of these supercentenarians have revealed that they live so long because they are able to escape major causes of death, such as vascular-related diseases, diabetes or metastatic cancer [5]. A key factor in avoiding or recovering from severe diseases is having a potent immune system, i.e., a high immune competence [6]. These elderly individuals maintain a robust immune system, characterized by a sufficiently high number of naïve T and B cells and good levels of NK (natural killer) cells, while also having a low rate of chronic systemic inflammation [7]. As a result, healthy centenarians exhibit normal T cell numbers, increased antibody production and preserved cytotoxicity of CD8^+^ T cells and NK cells [8].

**Figure 1:**
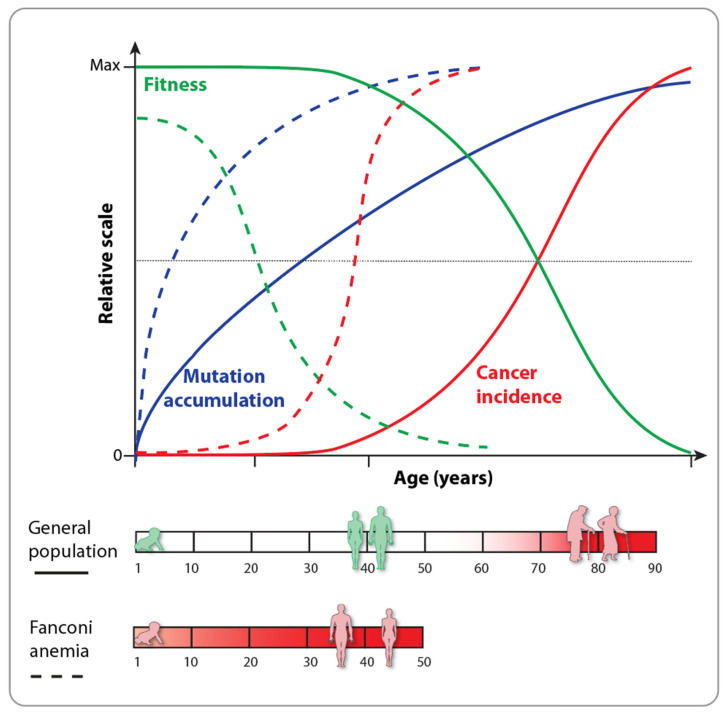
Comparing the lifespan of the general population to that of individuals with Fanconi anemia reveals distinct differences. In the general population (solid lines), overall body fitness begins to decline around age 45, coinciding with an increased rate of cancer. In contrast, for individuals with Fanconi anemia (dashed lines), these curves are shifted to the left, indicating that these changes occur much earlier. This shift is indicative of a premature-aging disease, resulting in a lower life expectancy for the individuals concerned.

Like the abilities to proliferate and differentiate, the processes of aging and death are inherent to the approximately 400 different tissues and cell types that make up the human body [9]. A fundamental evolutionary principle of multicellular organisms is to pass their genes to the next generation, necessitating reproduction [10]. In the general human population, the lifespan includes a period of growth and differentiation that culminates in sexual maturity, marking the peak of fitness and fertility. This is followed by a phase of aging characterized by a loss of function at various levels of cells, tissues and the organism as a whole (Figure 1). After a relatively long period until sexual maturity (12–15 years) and an additional 20–30 years of childcare, the first 45 years of life are generally free of non-communicable diseases, such as cancer or diabetes. However, beyond this age, there is no selective mechanism to prevent the onset of disease. Consequently, the incidence of age-related, non-communicable diseases increases significantly, contributing to the decline in overall fitness and the acceleration of the aging process [11].

The understanding of the molecular and cellular basis of aging has greatly advanced through the study of syndromes and diseases that represent malfunctions of normal processes [12]. For example, in individuals with premature-aging diseases like Fanconi anemia (Section 2), the high rate of stem cell exhaustion at birth, the increase in cancer incidence and the overall decrease in fitness occur decades earlier compared to the general population (Figure 1). Thus, Fanconi anemia and other premature-aging diseases like Hutchinson–Gilford progeria syndrome, Werner syndrome, ataxia–telangiectasia, Bloom syndrome, Nijmegen breakage syndrome, xeroderma pigmentosum and dyskeratosis congenita act as time-lapses of aging, resulting in a lower average lifespan [13]. The primary aging hallmarks, “genomic instability” and “epigenetic alterations”, appear to be the main drivers of these premature-aging syndromes. However, the different clinical presentations of these diseases suggest that other hallmarks, such as “deregulated nutrient sensing” (Section 3), modulate the process of accelerated aging [14].

In this review, we will use Fanconi anemia as a principal example of a premature-aging disease to illustrate that aging results from an imbalance between endogenous repair mechanisms and the accumulation of both exogenous and endogenous damage. We will discuss the impact of lifestyle choices (Section 4), such as a healthy diet and sufficient physical activity, to understand the nutrigenomic principles underlying aging. This insight will not only benefit individuals with Fanconi anemia but also the general population, paving the way for both prevention strategies and treatment options for aging and age-related diseases.

## 2. Fanconi Anemia and the Hallmarks of Aging

A common characteristic of the more than 100 known premature-aging syndromes is that they lead to accelerated aging [15]. These syndromes often present clinical features such as cancer, CVD and neurodegeneration, which occur at a much younger age and progress more rapidly than in the general population. Most premature-aging syndromes are caused by defects in genes that lead to genomic instability [16]. Postmitotic neurons are particularly sensitive to defects in DNA repair, resulting in many of these syndromes displaying a neurodegenerative phenotype [17]. Unlike the laminopathy Hutchinson–Gilford progeria syndrome [18], most DNA repair deficiency-based premature-aging syndromes are recessive, meaning that both alleles of the respective gene need to be mutated [19].

Fanconi anemia was defined nearly 100 years ago by Guido Fanconi as an anemia [20], primarily because the majority of affected individuals experience progressive bone marrow failure in the first decade of life [21]. In the general population, bone marrow failure is typically observed only in elderly individuals [22], which strongly suggests that Fanconi anemia should be considered a premature-aging disease. Fanconi anemia is a segmental premature-aging disease, affecting only certain tissues, such as the bone marrow and epithelial linings exposed to atmospheric oxygen, including the oral mucosa and the anogenital tract [23]. The functional decline in these tissues is primarily due to underlying defects in the DNA repair machinery. There are currently 22 known *FANC* genes that cause Fanconi anemia, all of which encode proteins involved in resolving DNA interstrand crosslinks [24]. The inactivation of these genes leads to the accumulation of double-strand breaks and genomic instability. Initially, this results in a high turnover of hematopoietic stem cells (HSCs), and eventually, their exhaustion. Consequently, most patients with Fanconi anemia require bone marrow transplants from healthy donors [25,26]. Interestingly, supra-pharmacological doses of testosterone analogs like danazol and oxymetholone have been shown to be effective in stabilizing or even improving blood cell counts in these patients [27,28,29,30].

Nine of the *FANC* genes encode components of a ubiquitin ligase complex, known as “upstream genes,” which activate “downstream genes” that encode tumor suppressor proteins, such as BRCA1 (BRCA1 DNA repair associated), BRCA2, BRIP1 (BRCA1 interacting helicase 1) and PALB2 (partner and localizer of BRCA2) [31]. Individuals with Fanconi anemia, who have recessive mutations in downstream genes, exhibit a much more aggressive phenotype, with only a few surviving beyond 10 years of age [32]. Additionally, approximately 80% of individuals with Fanconi anemia display easily detectable malformations, such as absent or malformed thumbs, short stature, microcephaly, skin anomalies and malformations of internal organs, including missing kidneys, horseshoe kidneys, disruptions of the anogenital tract and heart defects [33]. The remaining 20% of individuals with recessive mutations in one of the 22 *FANC* genes have a milder phenotype and no or very mild malformations [34]. Interestingly, siblings with Fanconi anemia, i.e., identical mutations, can show significant differences in their clinical presentation [35,36]. This suggests that, to some extent, lifestyle and environmental factors (exposome) affecting the epigenome, such as changes in DNA methylation and histone modifications, play a critical role in the clinical manifestation and progression of Fanconi anemia. Therefore, the aging hallmark “epigenetic alterations” significantly impacts the disease phenotype.

**Figure 2:**
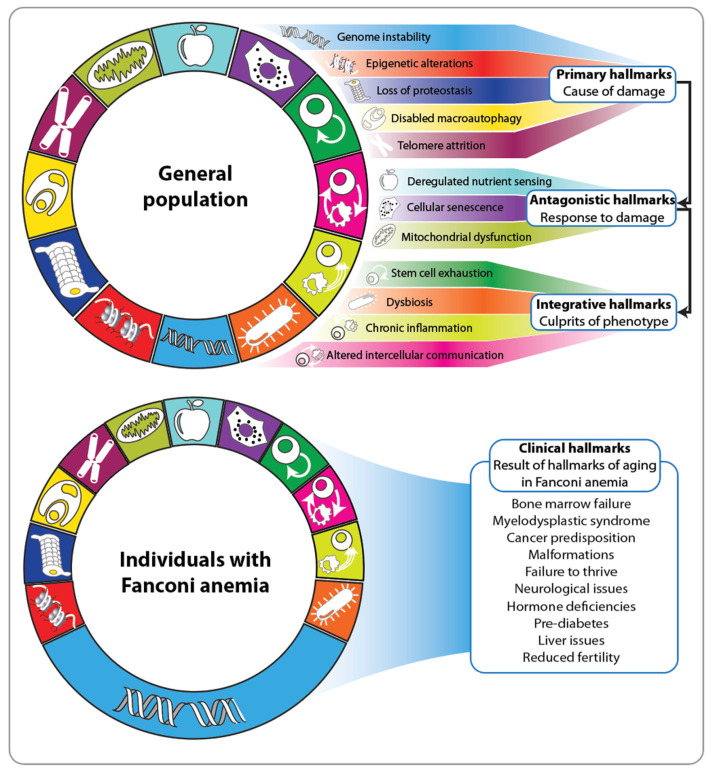
Hallmarks of aging in the general population (**top**) and in Fanconi anemia (**bottom**). Twelve major hallmarks determine the phenotype of aging, categorized into primary, antagonistic, and integrative groups. Typical clinical features of aging in Fanconi anemia are presented, bottom right.

Twelve different hallmarks form the molecular and cellular basis of aging in the general population [37] (Figure 2, top). They are categorized into three groups: primary (genome instability, epigenetic alterations, loss of proteostasis, impaired macro-autophagy and telomere attrition), antagonistic (deregulated nutrient sensing, cellular senescence and mitochondrial dysfunction) and integrative (stem cell exhaustion, dysbiosis, chronic inflammation and altered intercellular communication). The primary hallmarks create damage and are considered the root cause of aging [38]. In contrast, the antagonistic hallmarks counteract the primary ones and can have beneficial effects at low levels [39]. The integrative hallmarks predominantly emerge when the accumulated damage from the primary hallmarks cannot be mitigated by the antagonistic ones [37]. Interestingly, hallmarks such as impaired macro-autophagy, stem cell exhaustion, dysbiosis and chronic inflammation significantly impact the immune system [40]. For instance, a prominent aging-associated process is inflammaging, a proinflammatory phenotype that coincides with immunosenescence [41]. Immunosenescence exacerbates the aging process at the whole-body level, leading to a decline in immunocompetence. This decline impairs the immune system’s ability to eliminate infectious agents, infected cells and transformed cells. Thus, a decreased potency of the immune system is critical for everyone but is particularly fatal for individuals with Fanconi anemia.

**Figure 3:**
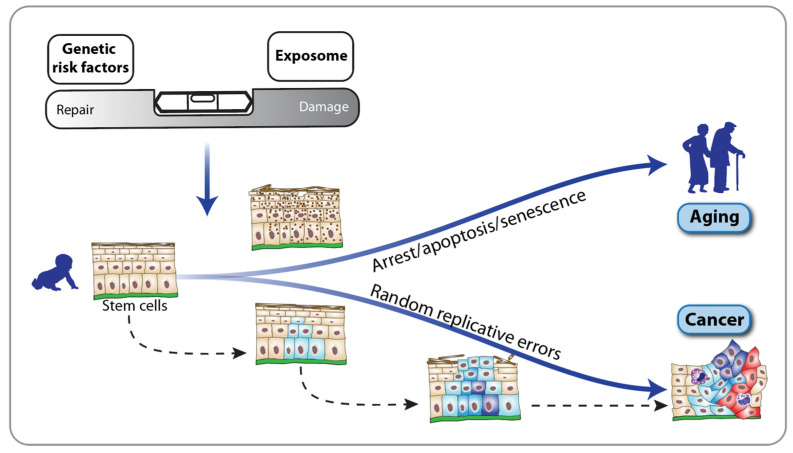
Interplay between aging and cancer. Genomic instability arises from an imbalance between DNA damage and the body’s intrinsic DNA repair mechanisms (**top**). This imbalance is influenced by both intrinsic factors, such as gene mutations, and extrinsic factors from the environment, including lifestyle choices related to diet, physical activity and smoking, collectively known as the exposome. When long-lived cells, like stem cells, accumulate DNA damage, they may undergo growth arrest, apoptosis or senescence or they replicate with random errors. This results in impaired tissue homeostasis and regenerative capacity, contributing to aging (**center**) and age-related diseases, like cancer (**bottom**).

Due to the underlying defect in the DNA repair machinery, the hallmarks of aging in Fanconi anemia are predominantly characterized by genome instability (Figure 2, bottom). This is clinically evident from the significantly increased risk, which is several hundred times higher than that in the general population, especially of developing squamous cell carcinoma in the oral cavity and anogenital tract at a much younger age (20–30 years) [23,42]. Of note, this cancer type, in the general population, is highly associated with unhealthy lifestyle choices over many years and thus a constant high burden of damage (Figure 3, top). Consequently, the lives of individuals with Fanconi anemia can only be effectively prolonged through early detection of precancerous stages and stringent surveillance [43]. The stepwise development of squamous cell carcinoma in accessible tissues like the oral cavity not only facilitates in vivo investigation of tumorigenesis but also provides multiple opportunities for prevention studies [44]. The accumulation of mutations in cancer driver genes, such as oncogenes and tumor-suppressor genes, is driven by genetic risk factors like impaired DNA double-strand break repair, as well as exposure to environmental factors, including those derived from diet (Figure 3, top). For example, fungal infections such as Candida albicans can lead to dysbiosis of the oral microbiome and altered intercellular communication [45]. These processes are integrative hallmarks of aging (Figure 2) and significant drivers of tumorigenesis in Fanconi anemia. Importantly, early detection and treatment of Candida albicans infections can reduce oral cavity dysplasia in individuals with Fanconi anemia [46,47]. Additionally, standard risk factors for developing squamous cell carcinoma, such as the use of tobacco and alcohol, should be avoided by Fanconi anemia patients. While these lifestyle recommendations are beneficial for everyone, they are particularly crucial for individuals with Fanconi anemia.

The progression from a healthy, non-transformed cell to low-grade and high-grade dysplasia and cancer follows a multi-step process in the tumorigenesis of all solid cancers [48] (Figure 3, bottom). This process can span decades in the general population but may occur within months to a few years in individuals with Fanconi anemia. At the molecular level, this multi-step tumorigenesis is marked by the accumulation of genomic and epigenomic changes that endow cells with new capabilities, such as self-sufficiency in growth signals, resistance to antigrowth signals, tissue invasion and metastasis, which are collectively known as the hallmarks of cancer [2,49]. These properties enable transformed cells to survive, proliferate and spread. In the general population, the hallmarks of cancer can develop in various sequences, while in individuals with Fanconi anemia, defective DNA repair pathways and the resulting genome instability are always the initial triggers [50]. The fact that some hallmarks of cancer, such as genome instability and epigenetic alterations, also serve as hallmarks of aging highlights the close connection between aging and tumorigenesis [3]. Along the timelines of both processes, there are critical bifurcation points where cells may either enter senescence, i.e., preferring to age and die, or proceed with tumorigenesis, achieving a state of unchecked proliferation (Figure 3). On the level of organs or the whole body, aging can be viewed as a cancer protection mechanism [51]. Accordingly, individuals with Fanconi anemia, who have a significantly higher risk of developing cancer, also experience accelerated aging compared to the general population. Thus, the heightened cancer predisposition in Fanconi anemia underscores its classification as a premature-aging disease.

## 3. Nutrigenomic Principles and Fanconi Anemia

Nutrient-sensing pathways are fundamental to the aging process, and their deregulation is one of its hallmarks (Figure 2). Food intake activates pathways that stimulate various physiological processes, including reproduction, which is often compromised by a limited lifespan [52]. Conversely, under conditions of starvation or when the nutrient-sensing pathways are genetically disrupted, reproduction is delayed, and lifespan increases [53]. Thus, food availability influences the speed of aging, at least in model organisms. Interestingly, most individuals with Fanconi anemia have a short stature and are known as “picky eaters” because their hormonal response to food intake and ghrelin secretion are disrupted [54]. This distraction is clinically represented by endocrine dysfunction, insulin resistance, and type 2 diabetes (T2D) occurring at a young age in these individuals [55] and delayed puberty. Dietary restriction, involving a reduction in daily caloric intake by 20–40% without causing malnutrition, has been shown to counteract aging, whereas anabolic signaling accelerates it [11]. For example, dietary restriction can increase the lifespan of rodents by up to 60%. Generally, dietary-restricted rodents exhibit various metabolic, hormonal and structural adaptations, such as higher insulin sensitivity, reduced inflammation and decreased oxidative damage [56]. Similar benefits are observed in dietary-restricted rhesus monkeys, which show a reduced incidence of cancer and cardiovascular diseases [57]. In humans, dietary restriction also provides benefits against obesity, insulin resistance, inflammation and oxidative stress [58]. Humans on dietary restriction display hormonal adaptations, such as increased levels of adiponectin and reduced concentrations of T3 (triiodothyronine), testosterone and insulin. Additionally, lower concentrations of cholesterol and the inflammatory marker CRP (C-reactive protein) are observed, along with reduced blood pressure. Thus, in rodents, monkeys and even humans, dietary restriction offers protection against age-related diseases. This leads to the hypothesis that the reduced food intake in individuals with Fanconi anemia is an intrinsic response to their premature-aging phenotype.

**Figure 4:**
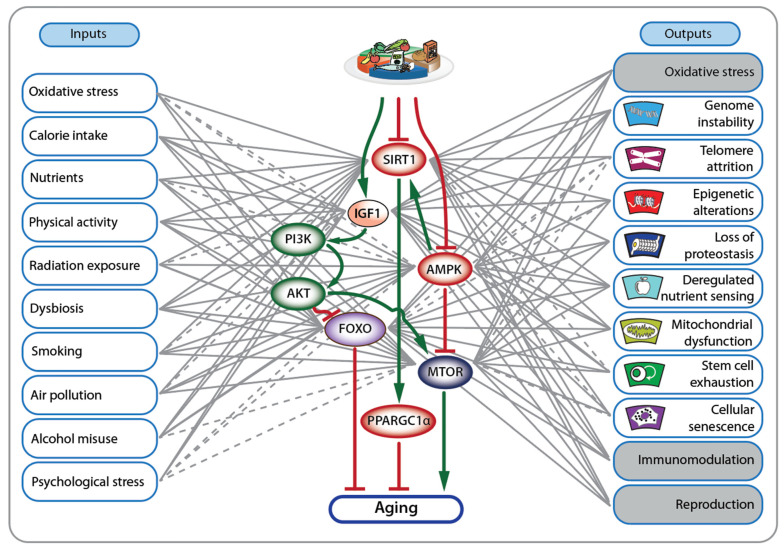
Nutrient-sensing and aging pathways. Signal transduction pathways involving pivotal proteins, such as IGF1, AMPK, SIRT1, FOXO, MTOR and AMPK (**center**), respond to a variety of input signals (**left**) and activate numerous pathways that regulate various hallmarks of aging and related physiological functions, including immunomodulation and reproduction (**right**). The overarching outcome of these signaling processes is that high food intake promotes aging, whereas dietary restriction promotes longevity. Solid lines denote established connections, while dashed lines suggest links requiring further confirmation.

A central sensor of low energy intake or fasting is the kinase AMPK (adenosine monophosphate-activated protein kinase), which is activated by a high intracellular AMP/ATP ratio [59] (Figure 4, center). Similarly, low energy states are sensed by sirtuins (SIRTs), which are activated by the electron transporter NAD^+^ (nicotinamide adenine dinucleotide) [60]. Since SIRT enzymes are part of the histone deacetylase family, diet-induced changes in their activity directly impact chromatin, the physical manifestation of the epigenome [6]. In contrast, food intake in mammals is sensed via the peptide hormone GH1 (growth hormone 1), its secondary mediator IGF1 (insulin-like growth factor 1), and insulin [61]. IGF1 and insulin activate a central signal transduction cascade involving the kinases PI3K (phosphatidylinositol-4,5-bisphosphate 3-kinase) and AKT (AKT murine thymoma viral oncogene homolog) via their specific membrane receptors IGF1R (IGF1 receptor) and INSR (insulin receptor) [61]. Central downstream effectors of AKT, AMPK, and SIRT1 include the transcription factor FOXO1 (forkhead box O 1), the kinase MTOR (mammalian target of rapamycin) and the coactivator protein PPARGC1α (peroxisome proliferator-activated receptor gamma coactivator 1A), respectively. FOXO1 and PPARGC1α inhibit aging, while MTOR promotes it [9]. These central signaling proteins not only sense the energy status of cells but also respond to numerous other input signals, such as oxidative stress, physical activity, radiation exposure, dysbiosis, smoking, air pollution, alcohol misuse and psychological stress (Figure 4, left). The outputs of this signal transduction network include many hallmarks of aging, such as genome stability, telomere attrition, epigenetic alterations, loss of proteostasis, deregulated nutrient sensing, mitochondrial dysfunction, stem cell exhaustion and cellular senescence, as well as oxidative stress response, immunomodulation and reproduction (Figure 4, right). In effect, in response to cellular damage, food shortage or other stresses, the defensive strategy of minimizing cell growth and metabolism enables the body to survive longer. Accordingly, many clinical features of Fanconi anemia can be reinterpreted from the perspective of this stress response.

To extend the lifespan of individuals with a premature-aging condition like Fanconi anemia, as well as the general population, it is crucial to activate the AMPK-SIRT1-FOXO1 signal transduction network positively, while minimizing activation of the IGF1/insulin-PI3K-MTOR pathway. For instance, although inhibiting the MTOR pathway with rapamycin ideally promotes longevity, its broad impact can lead to side effects such as impaired wound healing and insulin resistance [62]. Interestingly, MTOR plays a role in sensing cellular amino acid levels within a complex associated with the lysosome. Consequently, restricting dietary protein or specific amino acids like methionine can promote healthy aging [63]. Notably, legumes and other plant-based proteins have very low methionine levels compared to animal sources. However, evidence supporting protein restriction in the general population varies depending on age and type of protein. Nevertheless, substituting animal protein with plant-based alternatives is likely to foster healthy aging [64] and should also benefit individuals with Fanconi anemia.

The deacetylase activity of SIRTs plays a critical role in counterbalancing nutrient-driven protein acetylation [65]. During fasting or exercise, NAD^+^ levels increase in skeletal muscle, liver, and adipose tissue, whereas a high-fat diet reduces the NAD^+^/NADH ratio. Pharmacologically targeting SIRTs, especially SIRT1, holds promise for treating T2D and potentially slowing down aging [66]. The search for natural or synthetic SIRT activators has identified several compounds, with resveratrol garnering significant attention. Resveratrol enhances mitochondrial activity and metabolic control in humans, potentially increasing healthspan. The activity of SIRT1 is closely intertwined with AMPK [67]. Both proteins can be activated by a healthy lifestyle, encompassing moderate food intake and adequate physical activity (Section 4). Notably, a plant-based diet, rich in polyphenols, such as flavonoids from vegetables, fruits, coffee, tea, soy and cocoa; lignans from grains, seeds, and vegetables; phenolic acids from nuts, coffee and fruits; and resveratrol from wine and grapes, can modulate AMPK and SIRT activity [68]. Furthermore, synthetic compounds like antidiabetic drugs (e.g., metformin, phenformin and thiazolidinediones) and plant-derived products (e.g., epigallocatechin gallate from green tea, capsaicin from peppers, curcumin from turmeric) activate AMPK [69]. However, their activation, including that of metformin and resveratrol, is typically indirect, involving increased cellular AMP and ADP levels that inhibit mitochondrial ATP synthase. Notably, metformin is recognized as a therapy for extending the lifespan of individuals with Fanconi anemia [70].

## 4. Healthy Aging and Longevity of Individuals with Fanconi Anemia

Healthy aging is defined as maintaining physical, mental and cognitive health as people grow older, while delaying or preventing the onset of frailty. Thus, healthy longevity means not only living longer but also extending the healthspan [71]. Healthy aging and longevity are influenced by a combination of genetic and non-genetic factors. However, like most non-communicable diseases, the genetic impact on longevity is estimated to be less than 25%. Therefore, for the general population, aging is primarily determined by environmental factors [72]. Consequently, there should be a greater focus on the substantial preventive potential of diet and physical activity to ensure individuals enjoy healthy years as they age. Interestingly, about 2500 years ago, it was recognized that food could be medicine, highlighting that the amount, type, and timing of eating are powerful and safe ways to improve health and extend longevity. More recently, it has become evident that nutrition should be tailored to an individual’s age, gender, genetic variations, and metabolic risk status, leading to the concept of precision nutrition [73].

Nutrition is essential for life, but the effects of nutritional molecules on health are complex and influenced by many factors. A diet comprises food groups that collectively meet the body’s needs for macro- and micronutrients. Besides nutrients, food contains hundreds of bioactive compounds that act as signaling molecules influencing metabolism [74]. These bioactive molecules can signal over long distances (endocrine signaling), act locally between neighboring cells (paracrine signaling), or communicate within the cell itself (autocrine signaling). The lipophilic fraction of these molecules, such as steroid hormones or eicosanoids like prostaglandins, can cross the plasma membrane and bind to transcription factors in the cytoplasm or nucleus [75]. In contrast, the larger hydrophilic fraction of signaling molecules binds to membrane proteins on the surface of target cells. Macronutrients, such as fatty acids, cholesterol, glucose, and amino acids, and micronutrients, such as vitamins A, D and E, calcium and iron, can act as ligands for nuclear receptors or as enzyme cofactors [76]. For example, vitamin D is known to directly affect immunocompetence via its nuclear receptor VDR, which, together with pioneer factors, modulates the epigenetic programming of the myeloid line of hematopoiesis [77]. Nutrients that bind to membrane proteins initiate intercellular signaling pathways that modulate the activity of transcription factors and chromatin-modifying enzymes. The target genes of these regulators encode proteins critical for nutrient transport, uptake, storage and metabolic pathway enzymes. The daily interaction between diet and genome modulates the gene regulatory networks in metabolic organs such as skeletal muscle, adipose tissue, pancreas and liver, as well as in the immune system and brain [78]. These molecular and cellular processes maintain the body’s homeostasis and prevent the onset of non-communicable diseases.

Individual nutrients or food groups have relatively small effects on human health, but the overall quality of a diet and the interactions among many nutrients are critical [79]. In this context, the impact of healthy dietary patterns, such as the Mediterranean or Nordic diet, is significant. The Mediterranean diet is associated with a reduced risk of obesity, T2D and CVD, as well as a slower progression of age-related cognitive decline, leading to a lower risk of dementia and Alzheimer’s disease [80]. Additionally, adherence to this diet is linked to reduced mortality and increased longevity. The Nordic diet is very similar to the Mediterranean diet, particularly in its emphasis on a plant-based diet of local origin. For example, instead of olive oil, rapeseed oil is used, while whole grain sources are rye, barley and oats. Adherence to Nordic diet is associated with reduced risk of CVD and T2D and total mortality leading to increased longevity [81].

**Figure 5:**
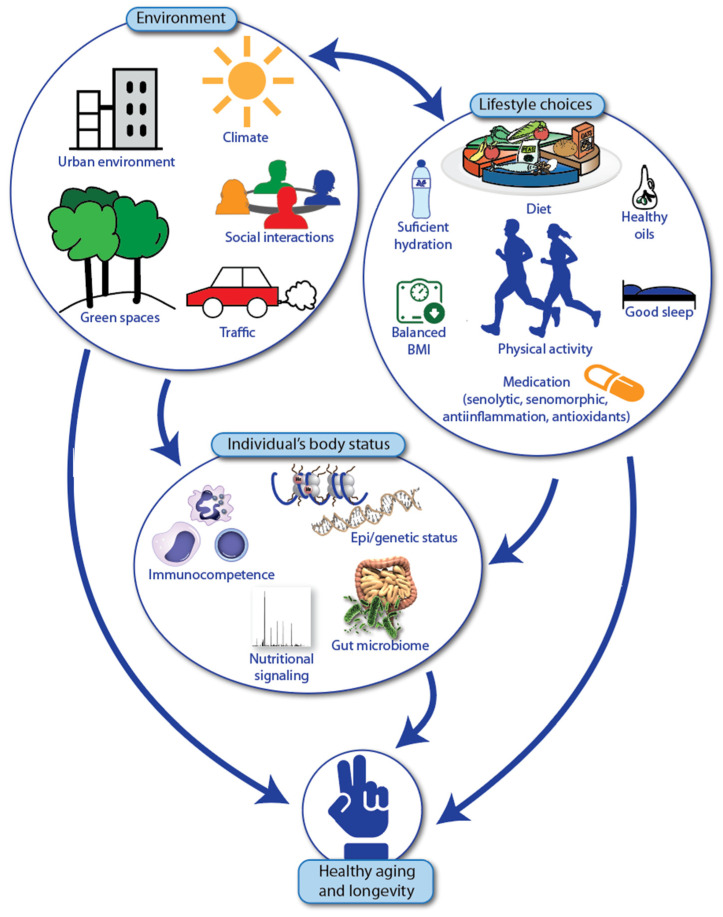
Interplay of external environment, lifestyle choices and body status in healthy aging and longevity. Observations from centenarians living in Blue zone areas indicated that healthy aging and longevity depend on a multitude of factors of the external environment (**top left**), various lifestyle choices (**top right**) and the status of the individual’s body (**bottom**).

Plant-based diets vary in composition, ranging from those that completely exclude animal products (vegan) to those that include a high consumption of vegetables. People who follow vegetarian diets have a lower risk of T2D, CVD and cancer [82]. Additionally, plant-based diets are associated with increased longevity due to lower overall mortality. However, a vegan diet may lack important nutrients, such as vitamin B12, vitamin D_3_, iron, calcium and long-chain omega-3 fatty acids. The Japanese island of Okinawa is one of the so-called Blue Zones, where a high number of centenarians live [83]. The diet of Okinawans consists mainly of root vegetables, yellow and green vegetables, seaweeds and algae, soybean-based foods, tea and a variety of medicinal plants, with animal products making up only 1% of their diet. Occasional consumption of meat or animal products is also characteristic of other Blue Zone areas, such as Sardinia (Italy), the Nicoya Peninsula (Costa Rica), Ikaria (Greece) and Loma Linda (CA, USA). In addition to their plant-based diets and moderation in food consumption, these centenarians engage in high levels of physical activity and maintain strong familial and social connections. Taken together, there is convincing evidence that healthy aging and longevity are promoted by a combination of a plant-based diet, physical activity and strong social ties [1] (Figure 5).

Taken together, there is convincing evidence that healthy aging and longevity are promoted by [84]:

- A plant-based diet composed of fruits, vegetables, leafy greens (foods rich in polyphenols, antioxidants, folate, fibers, potassium, etc.), nuts and seeds, such as walnuts, flaxseed and rapeseed oil (foods rich in the essential ω-3 fatty acid α-linolenic acid);
- Fish and fish oil (containing the marine ω-3 fatty acids eicosapentaenoic acid and docosahexaenoic acid);
- Physical activity, normal-range BMI (body mass index) and low alcohol intake;
- Avoiding saturated fatty acids (animal products, palm oil, coconut oil) and trans-fatty acids (hardened fats).

Thus, the recommendation for the so-called healthy eating plate [79] is the following:

- Enjoy vegetables, fruits, whole grains, beans, legumes, nuts, plant-based proteins, lean animal proteins, skinless poultry, fish and seafood.
- Limit sweetened drinks, alcohol, sodium, red and processed meats, refined carbohydrates like added sugars and processed grain foods, full-fat dairy products, highly processed foods, tropical oils like coconut and palm.
- Avoid trans-fats and partially hydrogenated oils.

For example, ketogenic diets (1 g of protein per kg of body weight, less than 15 g of carbohydrates/day and the rest coming from fat) are very low in carbohydrates and induce ketosis, i.e., a condition in which energy for the body is primarily provided by short chain fatty acids, referred to as ketone bodies [85]. Interestingly, the majority of individuals with Fanconi anemia are lean and seem to be in a status of lipolysis, which resembles a ketogenic diet [86]. This is another example of a dietary adaption of Fanconi individuals, in order to counteract their premature-aging status.

In summary, our recommendations for individuals with Fanconi anemia on how to slow down the aging process are similar to those for the general population: make healthy lifestyle choices concerning diet, physical activity and sleep. Additionally, maintain a network of positive social interactions and avoid stress caused by an unhealthy environment and climate. When these recommendations are not sufficient, medications such as senolytic, senomorphic, anti-inflammatory and antioxidant compounds (e.g., metformin or quercetin) may be considered (Figure 5).

## 5. Conclusions

The topic of aging concerns everyone, but individuals with premature-aging diseases like Fanconi anemia experience its effects far more drastically. Aging is not a disease but represents natural changes in physiological and biochemical processes in the human body. These changes significantly affect the risk of developing various non-communicable diseases. Interestingly, premature-aging syndromes, with cancer as the main cause of death, are all associated with defects in genomic maintenance and display clinically a variable severity of immunodeficiency. Also, in Fanconi anemia the most affected hallmark is genomic instability, followed by epigenetic alteration and altered nutrition sensing. Both the general population and individuals with Fanconi anemia differ in their rates of aging. Although there is a genetic basis for longevity, lifestyle choices such as dietary habits, smoking, physical inactivity and many environmental factors play a significant role.

The molecular mechanisms underlying these non-genetic factors involve cellular disturbances that modulate signal transduction pathways, thereby influencing the epigenome through the regulation of chromatin-modifying enzymes. Epigenetic changes play a pivotal role in aging and are considered one of its hallmarks. The epigenome retains the impact of environmental influences through alterations in DNA methylation, histone modifications, and the three-dimensional organization of chromatin. Changes in the epigenome, particularly in the DNA methylome, correlate with chronological age and age-related diseases like cancer. At a specific chronological age, some individuals may exhibit a “younger” epigenome in their tissues, while others may display an “older” epigenome [87]. Those with an older epigenome tend to experience earlier onset of age-related diseases and may succumb at a younger chronological age; a pattern observed in individuals with premature-aging syndromes. Conversely, the offspring of supercentenarians often exhibit a lower epigenetic age in their blood compared to age-matched controls, making epigenetic signatures valuable biomarkers of aging. For individuals with Fanconi anemia, this holds promising future opportunities. Although the majority of recommendations for changes in the exposome are the same as for the general population, the accelerated nature of aging in these individuals allows for the effects to be monitored on a molecular level. This monitoring can guide personalized recommendations, forming a fundamental basis for precision nutrition and precision prevention. Additionally, since these epigenetic signatures are protein-based, they could potentially serve as druggable targets, offering opportunities to delay or even reverse age-related diseases such as cancer. Therapeutic and preventive strategies developed for premature-aging syndromes may also be applicable to the general population.
